# Pancreaticoduodenectomy for hepatic portal lymph node metastasis after hepatic resection for hepatocellular carcinoma: A clinical case report

**DOI:** 10.1016/j.ijscr.2021.105921

**Published:** 2021-04-27

**Authors:** Tran Que Son, Tran Hieu Hoc, Vu Duc Long, Nguyen Toan Thang, Tran Thu Huong, Pham Van Tuyen

**Affiliations:** a Department of Surgery, Hanoi Medical University, Viet Nam; b Emergency Center (A9), Bachmai Hospital, Viet Nam; c Department of anesthesia, Hanoi Medical University, Viet Nam; d Department of Pharmacy, Bachmai Hospital, Viet Nam; e Center for Pathology and Cytology, Bach Mai Hospital, Viet Nam

**Keywords:** Case report, Pancreaticoduodenectomy, Lymph node, Pancreatic head, Metastasis, Liver resection, Hepatocellular carcinoma

## Abstract

**Introduction:**

In 2018, Hepatocellular carcinoma (HCC) was predicted to be the sixth most commonly diagnosed cancer. Extra-hepatic metastasis due to HCC is a poor prognostic factor, depending on the stage of the disease.

**Presentation of case:**

We report a case of a 52-years old male who had undergone Segment 5 (S5) hepatectomy for HCC of 4.7 × 2 cm. Transcatheter arterial chemoembolization (TACE) four times postoperatively was performed based on a preoperative diagnosis of a recurrent tumour at the S1. After 2 years, the solitary tumour (7.5 × 2.5 × 3.5 cm) is located behind the right lobe of the liver and the head of the pancreas. The tumour was abnormally supplied with blood from the superior mesenteric artery (SMA) and the gastroduodenal artery (GDA). The patient was underwent pancreaticoduodenectomy (PD) to remove a large tumour. Postoperative pathology and immunohistochemical staining showed metastatic HCC. There was no tumour recurrence after 6 months.

**Discussion:**

The organs in the body that liver cancer cells most often spread to are the lungs (44%), the portal vein (35%), the hepatobiliary ganglion (27%), and a small number of cases of bone, eye socket, bronchus metastases. Otherwise, recurrence of lymph nodes (LNs) after hepatectomy for HCC is very rare.

**Conclusions:**

HCC can metastasize to the hepatic pedicle LN after hepatectomy and maybe confused with recurrent liver tumours in the S1. Indications for PD are feasible for solitary metastatic at peri-pancreas. Pathology incorporating immunohistochemistry can determine the origin of metastases.

## Highlights

• Recurrence of lymph nodes after hepatectomy due to HCC is very rare at only approximately 1% to 2.5%.
• HCC can metastasize to the hepatic pedicle lymph node after hepatectomy and is easily confused with recurrent liver tumours in the S1
• Indications for pancreaticoduodenectomy are feasible for solitary metastatic at peri-pancreas

## 1 Introduction

Hepatocellular carcinoma (HCC) was predicted to be the sixth most commonly diagnosed cancer and the fourth leading cause of cancer death worldwide in 2018, with approximately 841,000 new cases and 782,000 deaths annually related to hepatitis B and C infections (1,2). HCC usually metastasizes via the bloodstream and, a lymphatic system to the lungs, adrenal glands, and bone (1,3). Besides, some rare cases involve orbital, mediastinal LNs, subclavian lymph node, skin, and jaw bone metastases ([4], [5], [6], [7], [8]). The rate of LNs metastasis for HCC ranges from 23.5% to 43.9%. However, LNs metastasis after hepatectomy is very rare, at only about approximately 1% to 2.5% depending on the study (3,6). Meanwhile in LNs metastasizes rates for colorectal cancer, extra-colorectal cancer, and intrahepatic biliary cancer are 14%, 40%, and 40% respectively (9). Regional LNs metastasis is a negative prognostic factor for long-term postoperative survival (5,9,10).

**Fig. 1:**
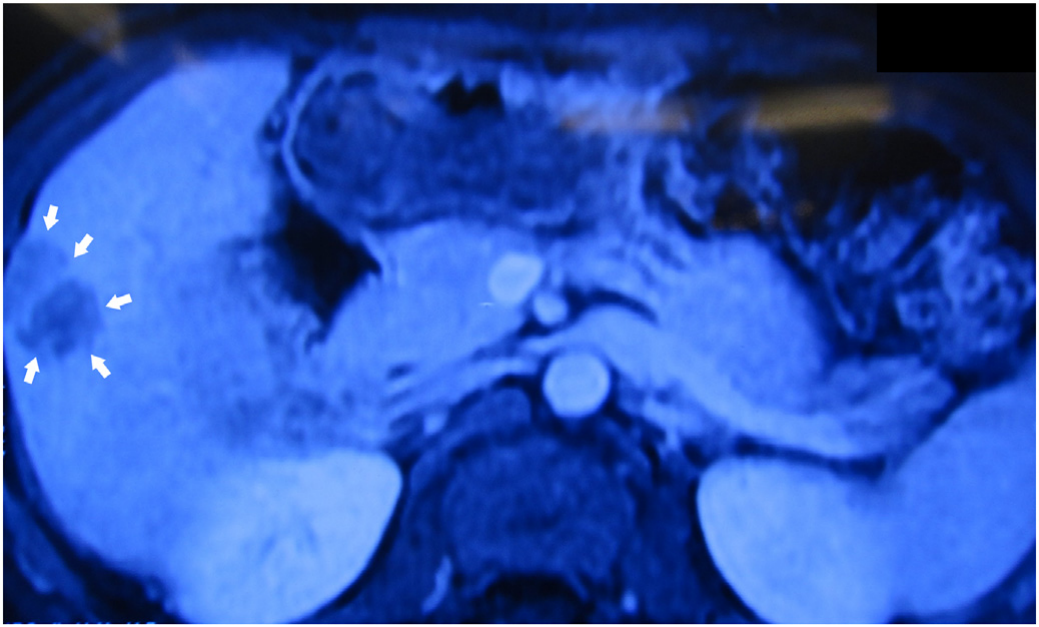
Liver tumour on Magnetic resonance imaging 29 × 22 mm (white arrow).

**Fig. 2:**
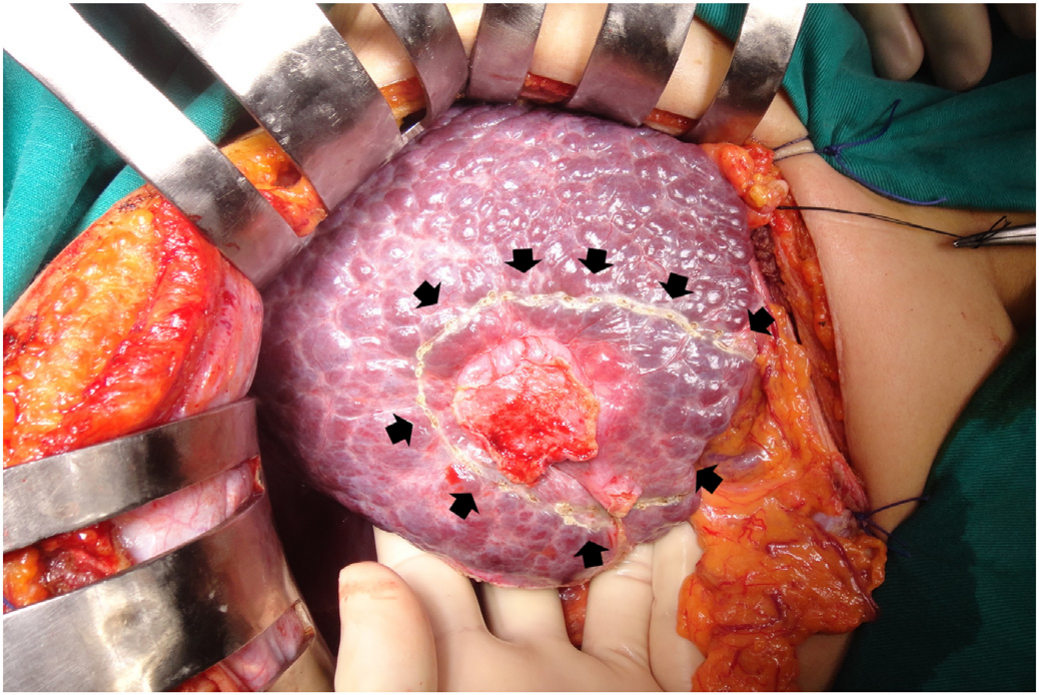
The liver tumour located in the segment 5 attached to the abdominal wall after 2 times TACE therapy.

**Fig. 3:**
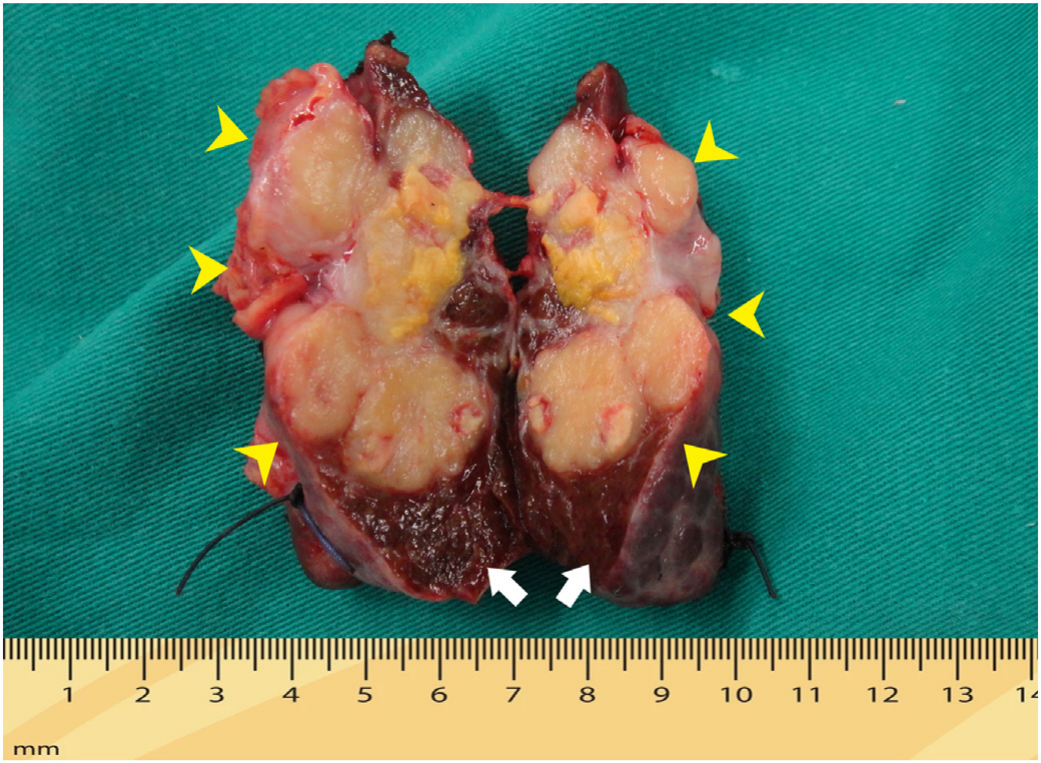
Liver tumour specimens for HCC after the first S5 resection. Healthy liver parenchyma (white arrow), tumour about 2 × 4.7 cm size (yellow pointed arrow). (For interpretation of the references to colour in this figure legend, the reader is referred to the web version of this article.)

**Fig. 4:**
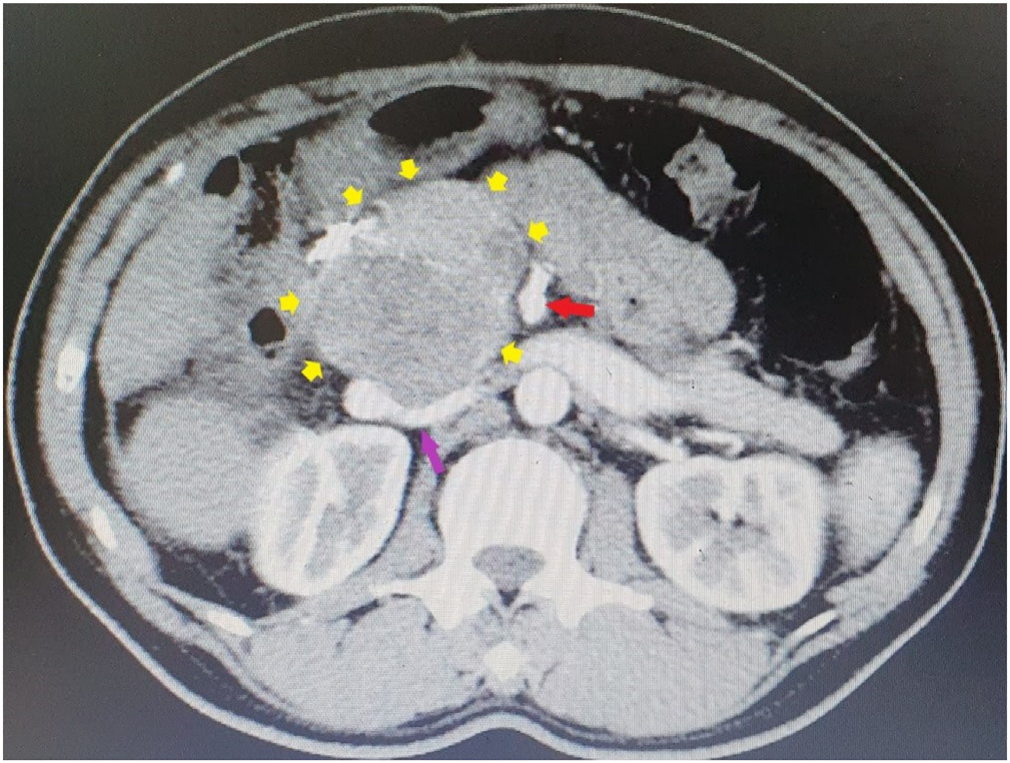
Computed tomography image of posterior large lymph node (yellow arrow). Upper mesenteric artery (red arrow), right renal artery (pink arrow). The lymph node metastatic close to the S1r position with size of 34 × 60 mm. (For interpretation of the references to colour in this figure legend, the reader is referred to the web version of this article.)

**Fig. 5:**
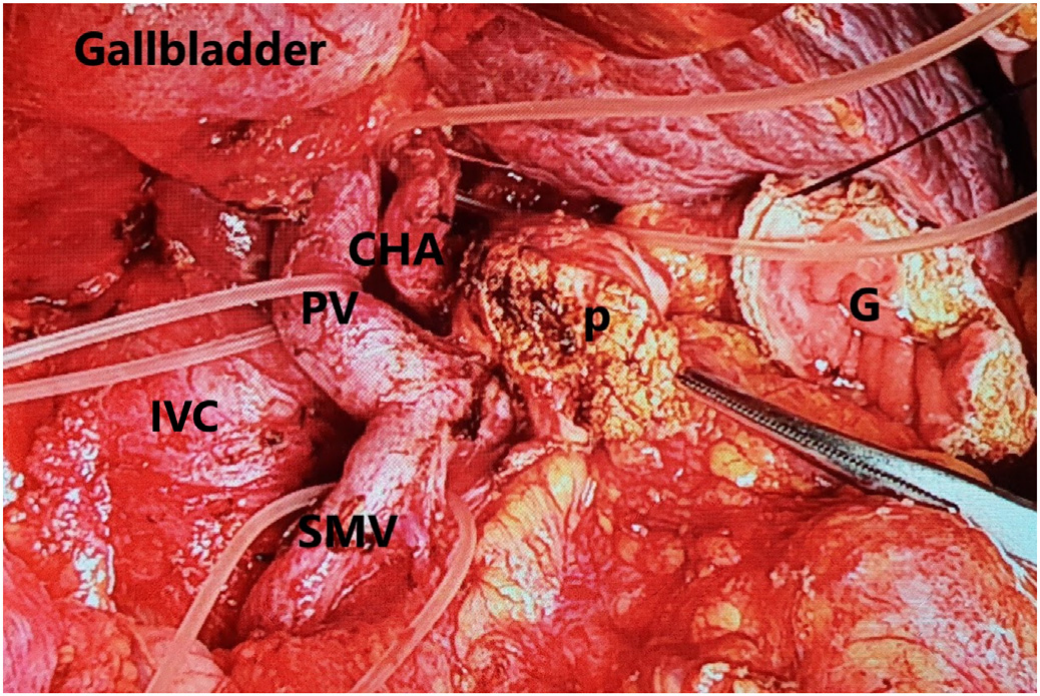
Pancreaticoduodenectomy and radical systematic lymph node. (PV portal vein, SMV superior mesenteric vein, G gastric, P pancreas remain).

**Fig. 6:**
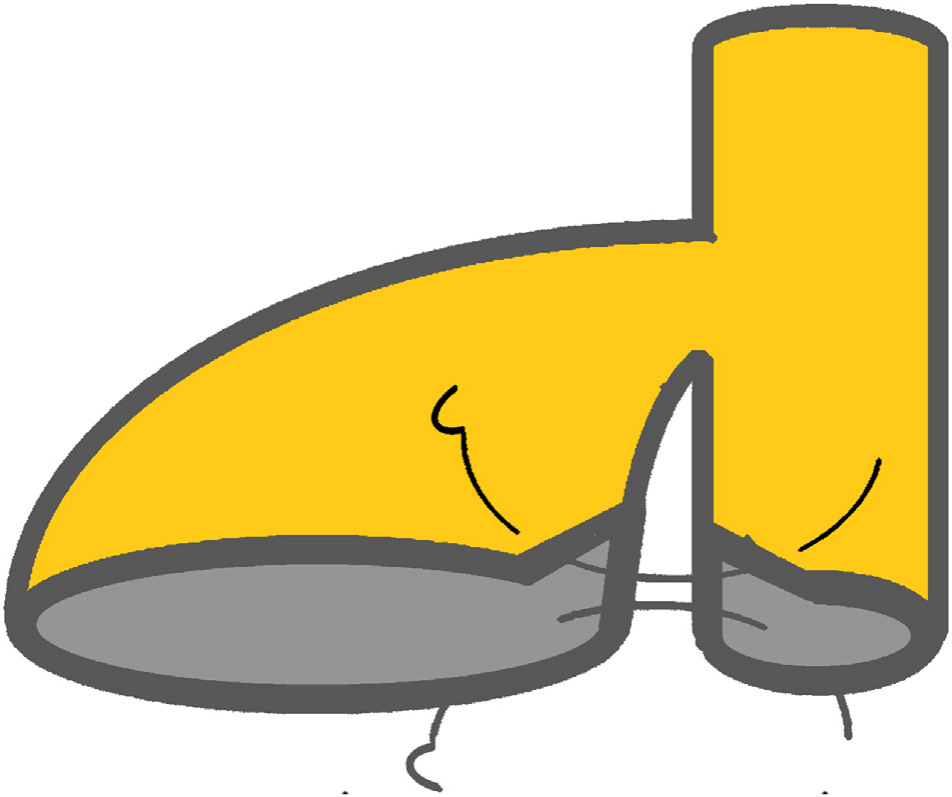
The image demonstrating how the neighboring walls of the gallbladder neck and CBD joined by two interrupted suture. Two sutures joined side wall of the CBD to the gallbladder neck and the sketch of new biliary size with 2 cm wide.

**Fig. 7:**
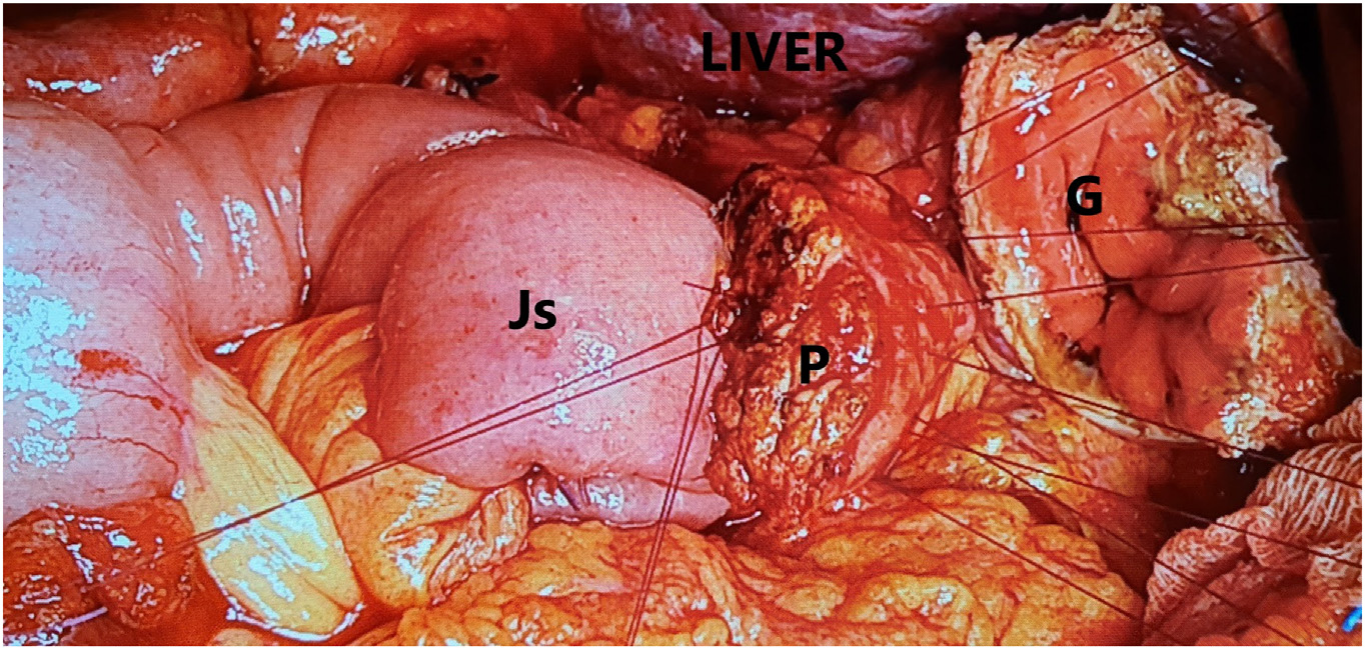
Blumgart anastomosis for pancreaticojejunostomy with 2 layers. Inner layer was duct-to-mucosa anastomosis which was constructed using interrupted sutures. Outer layer was four transpancreatic U-sutures were placed straight through the pancreatic remnant about 1 cm distal from the cut end (Monosyl 4–0, Ethicon). *Js Jejunal stump, P pancreas, G gastric stump.*

Staging and treatment regimens for primary liver cancer have been updated according to AASLD guidelines (11). Nevertheless, guidelines for the treatment of extra-hepatic recurrent HCC are limited and depend on the location (3,9,10). Surgery to remove metastatic tumours after liver resection is controversial, mostly being indicated in certain cases when the tumour is solitary (10). The 5-year survival rate for patients with LNs metastasis is approximately 20%, but resection of the affected LNs offers the best chance of long-term survival (12). When the tumour metastasizes to the hepatobiliary pedicle region or peripancreas head, PD procedure is necessary to ensure complete removal of the lesion. However, this is a complicated surgery due to complications and morbidity, especially in patients with a history of (Fig. 1, Fig. 2, Fig. 3, Fig. 4, Fig. 5, Fig. 6 and 7) surgery and cirrhosis. To the best of our knowledge, PD procedure due to HCC metastasis is very rare, with only 3 clinical cases in the literature to date (6,13).

We report a case of an old man who underwent a S5 hepatectomy for HCC. After 23.5 months, the patient underwent PD to remove LN recurrence. This is the first clinical case reported in Vietnam. This report may be useful for both education and clinical practice purposes.

This case report was prepared following SCARE Guidelines 2020 (14).

## 2 Case presentation

We present a case of an 52 years old elderly man and had a history of chronic hepatitis B virus infection. The patient had no history of jaundice, fever, nausea, vomiting and allergies reactions. A liver tumour was detected 3 years prior, and TACE was performed 2 times (Fig. 1). On September 17, 2018, hepatectomy for S5 for HCC (29 × 22 mm)/cirrhotic liver Child-Pugh score A was performed (Fig. 2). After that, he continued to receive anti-hepatitis B virus treatment and was periodically monitored. A single tumour near S1 was detected at 12 months after liver resection, and the patient underwent 4 times TACE received doxorubicin (50 mg/m2) and cisplatin (50 mg/m2) infusions into the liver via the transhepatic artery (Fig. 3, Fig. 4).

At the clinical re-examination at 23.5 months post-operatively, parameters were as follows: BMI = 19.9 kg/m^2^. The patient explained right lower rib pain.

### 2.1 Diagnostic assessment

Laboratory measurements were as follows: red blood cells 6.2, platelets 191 G/L, white blood cells 7.04 G/L, prothrombin (%) 110, fibrinogen 4.27 g/L, glucose 6.6 mmol/L, creatinine 80 g/L, albumin 42.1 g/L, total bilirubin 7.9 μmol/L, GOT 38 U/L, GPT 20 U/L, total AFP 1.3 ng/mL, AFP-L3 < 0.5% (normal <10%), and PIVKA-II 19 mAU/mL.

Computed tomography (CT) showed that a solitary density reduction mass of 34 × 60 mm, located behind the head of the pancreas and the portal vein (Fig. 4). The tumour was supplied with blood by the gastroduodenal artery (GDA) and the superior mesenteric artery (SMA). The pancreas was of a normal shape and size, without the pancreatic duct dilation (Fig. 4).

### 2.2 Therapeutic intervention

A second surgical procedure was performed on 4/9/2020. The operation was performed by a hepatobiliary surgeon with more than 7 years of experience and two resident doctors at Bach Mai Hospital - one of the largest hospitals in Vietnam.

The liver was cirrhotic, and no superficial metastases were found. The tumour was completely located under the right hepatic lobe, behind the portal vein and infiltration to the pancreatic head. Therefore, we decided to PD procedure standardized lymphadenectomy, and reconstruction with Child method instead of LN resection (Fig. 5). Especially, the common bile duct was not dilated. Therefore, we applied a pedicled gallbladder flap which designed by Jiquiao Zhu (15) as follows: after the dissection of the extrahepatic bile duct, the gallbladder was carefully dissected off the bed of the liver while preserving the cystic artery, then transected at the upper part of the gallbladder neck to achieve a large of 1.5 cm diameter. After that, the common bile duct (CBD) was transected equal to the gallbladder neck in length. The neighboring walls of the gallbladder neck and CBD were cut axially in the middle and joined as a common channel using two interrupted stitches (4–0 monosyl; Ethicon) to prepare for anastomosis (Fig. 6).

Pancreaticojejunostomy was performed by modified Blumgart anastomosis with a stent plastic placed in the pancreatic duct (Fig. 7). Histopathological staining by two methods HE and PAS following results: radial R0, tumour size 7.5 × 2.5 × 3.5 cm, rich nucleus cells were arranged in clusters, a penetrating and erasing ganglion structure. Immune tissue staining results were as follows: Glypican3 (+), TTF1 (+) cytoplasm, CK19 (+), CK7 (−), CK20 (−), Hepar 1 (−), Arginase1 (−), and CD10 (+). The histopathology and immunohistochemistry were consistent with HCC metastatic.

### 2.3 Follow-up and outcomes

The patient was admitted to the hospital for 9 days and discharged with no post-operative complications.

After 6 months, the patient has been re-examined 2 times since postoperative including: clinical examination, blood test, CA19.9, AFP, computed tomography. Until now, the patient was still alive with no recurrence.

## 3 Discussion

HCC is one of the most common malignancies in the world. Mortality is high due to rapid recurrence and distal metastasis (3,11). According to statistics of the World Health Organization (WHO), Vietnam has the 3rd highest rate of men with liver cancer in the world, just behind Mongolia and Laos. In Vietnam, liver cancer ranks first among the most common cancers in men and fifth in women with a prevalence of 25,335 cases. The prevalence rate among men is 39/100,000, while for females is 9,5/100,000. The incidence rate is 23,2 per 100,000 people in both sexes (2). Death can even occur because of acute abdominal bleeding due to rupture of metastatic lymph nodes behind the pancreas (8). Some of the organs to which liver cancer often spreads are the lungs (55%), bones (38%), and adrenal glands (4,6,7). LN metastasis due to HCC is not uncommon, approximately 23.5% to 43.9% based on the results of postoperative dissection (1,4,7,13,16). However, the rate of LN metastasis in patients with liver resection is only approximately 1% to 2.2% (5,6).

Single LN recurrence at the hepatoduodenal ligament and/or peri-pancreatic head usually has a good prognosis. Utsumi et al. reported two cases of PD with a survival time of 25 and 27 months, but one patient had a liver tumour recurrence after surgery. The remaining patient lived longer than 27 months and this was a very rare liver cancer case (6). Wojcicki et al. reported one patient who was 28 years old had LN metastasis with cirrhosis and underwent a total of five abdominal operations (one to remove the pancreatic mass due to metastatic peripancreatic lymphadenectomy and one thoracotomy who survived for 114 months. The author concluded that HCC can be metastasize to the abdominal and mediastinal LN several months or years after liver resection. Therefore, patients post-liver resection should be followed up by abdominal, thoracic computed tomography, or systemic imaging (PET-CT). Metastasis to the head of the pancreas can be removed by PD procedure safely (13). Postoperative histopathology is essential to identify the nature of metastatic tumours.

Ercolani et al. found a rate of 7.5% lymph node invasion for HCC, 14.0% for colorectal metastasis, 40.0% for metastasis from other sites, and 40.0% for intrahepatic cholangiocarcinoma. The survival time of lymph node metastases after hepatectomy is highly variable (1). Survival after 1 year, 3 years, and 5 years is reportedly 39.3%, 7.4% and 4%, respectively (17,18). Authors have also argued that regional lymphadenectomy after liver resection is a safe surgery and should be common in practice (9).

HCC receives most of its blood supply from branches of the hepatic artery, accounting for its characteristic enhancement pattern: early arterial enhancement with early “washout.” Hence, small foci of HCC may be seen within a regenerative liver nodule as foci of arterial enhancement. Interestingly, in this case, the tumour was at a very close position to S9 (or S1r) according to Kumon (19). The tumours were supplied from branches of the GDA and SMA. It had some characteristic of HCC tumours such as “washout” sign. Thus, the TACE procedure was being performed reach to 4 times after the first hepatectomy. However, in this case, the tumour still increased in size after 24 months, with poor chemical infiltration. As tumours 7.5 cm in diameter are continuous with S1r and close to the pancreatic, it is difficult to confirm whether the exact origin of the tumour was from the liver, LN metastasis, or periampullary tumour (Fig. 4). Overall, TACE is not very effective and tumour progression and angiogenesis are factors in determining the indication for surgery. The midgut blood supply to the tumour confirms the importance of determining vascular anatomy - particularly when TACE is employed but which clearly had no effect in this case.

There were some ways for treatment, such as simple right liver resection, right hepatectomy + PD, excision of the pancreatic mass by PD, or mere LN removal. Preoperative biopsy under ultrasonic guidance or computed tomography may be applied to determine the nature of the tumour cells (malignant or benign). To our knowledge, when a large tumour is located behind the head of the pancreas, a biopsy is very difficult and dangerous because large blood vessels can be damaged, increasing the risk of metastasis in the abdomen. Recent studies using 18F-FDG PET/CT to identify extra-hepatic metastases have reported a sensitivity of up to 79%, with 83% detection of tumours >1 cm, and 13% detection of tumours less than 1 cm (5).

Treatment options for recurrent ectopic metastatic lesions remain controversial, mostly regarding the symptomatic treatment of the whole system with the drug sorafenib. Surgery to remove metastatic lesions beyond the liver is limited to single, localized lesions. Results show that the removal of metastatic LNs alone is beneficial in selected patients, even though the prognosis of long-term postoperative survival is limited when the tumour has spread beyond the liver. Therefore, options should be chosen according to each case (10).

The current patient had a large tumour located completely behind the hepatic pedicle and surrounded by important vascular structures, adhering to the superior mesenteric vein, portal vein, and the head of the pancreas but also mobilization. In particular, no signs of peritoneal metastasis or liver metastasis were observed. Therefore, this was a rare case in which resection was possible but required major and risky surgery, potentially involving bleeding, and pancreatic fistula (Fig. 5, Fig. 7). PD procedure for dissection of LN metastasis is more difficult than for the primary tumour which is located around the ampulla of Vater because it is large and infiltrate to the portal vein or superior mesenteric artery.

Limitations of the study: there were only 6 months of follow-up after the second operation. It is necessary to continue chemotherapy according to the regimen, follow up regarding signs of recurrence by ultrasound, tomography, or PET-CT, and monitor complications of the bile-intestinal anastomosis.

In conclusion: HCC may recur in the posterior portal vein and posterior lymph nodes. Although treatment for distant metastases is controversial, surgery should be carried out when the tumour is solitary. PD in this situation is similar to that for a periampullary Vater tumour. After surgery, the patient should continue to follow up to detect recurrence and metastasis.

## Abbreviations

PET-CT: Positron Emission Tomography and Computed Tomography
BMI: Body Mass Index
AASLD: American Association for the Study of Liver Diseases
AFP: Alpha-Fetoprotein

## CRediT authorship contribution statement

Case report concept and design acquisition of data, analysis and interpretation of data; Dr. Tran Que. Son: literature review, writing-original draft and editing; Asso.Prof.PhD. Tran Hieu Hoc: literature review, critical revision of the manuscript; Dr. Vu Duc Long: literature review, discussion; Dr. Nguyen Toan Thang: case description and discussion, resources; Pharmacist Tran Thu Huong: resources, edit English language; MD. Pham Van Tuyen: read pathology template.

## Declaration of competing interest

All authors have no conflict of interest about this study.
